# Does Intermittent Nutrition Enterally Normalise hormonal and metabolic responses to feeding in critically ill adults? The DINE-normal proof-of-concept study

**DOI:** 10.1016/j.clnu.2025.10.003

**Published:** 2025-10-30

**Authors:** Clodagh E. Beattie, Borislavova Borislava, Harry A. Smith, Michael T. Ambler, Paul White, Danielle Milne, Aravind V. Ramesh, Alexander Ferriman, Thomas Fisher, Charlotte Horsley, Sherena Jackson, Chloe Jubainville, Kate Lobo, Hannah Maxfield, Javier T. Gonzalez, James A. Betts, Anthony E. Pickering, Matt Thomas

**Affiliations:** a Intensive Care Unit, North Bristol NHS Trust, Bristol, UK; b Anaesthesia, Pain and Critical Care Research, School of Physiology, Pharmacology and Neuroscience, University of Bristol, Bristol, UK; c Centre for Nutrition, Exercise and Metabolism, University of Bath, Bath, UK; d Mathematics and Statistics Research Group, University of the West of England, Bristol, UK

**Keywords:** Circadian rhythm, Critical care, Enteral nutrition, Glucose, Insulin, Intermittent fasting

## Abstract

**Background and aims:**

For intensive care unit (ICU) patients fed via a nasogastric (NG) tube, current guidelines recommend continuous feeding through the day and night. Emerging evidence in healthy individuals shows that NG feeding in an intermittent diurnal pattern promotes phasic hormonal, digestive and metabolic responses vital for effective nutrition, though this has not been studied in the critically ill population. This proof-of-concept study aimed to compare the effect of diurnal intermittent versus continuous enteral feeding on hormonal and metabolic outcomes in ICU patients.

**Methods:**

We conducted a single-centre, randomised, open-label trial in the ICU. Adult ICU patients that were anticipated to require NG feeding for >48 h were randomised to an intermittent diurnal regimen (feeds at 8:00, 13:00 and 18:00), or continuous feeding with equivalent nutritional value, for 48 h. The primary outcome was peak plasma insulin within 3 h of delivering the first intermittent feed on the second study day, compared to the same time period in the continuous group. Secondary outcomes included feasibility, tolerability and metabolic profiles.

**Results:**

Thirty patients were randomised to intermittent (n = 13) or continuous (n = 17) feeding. Two patients in the intermittent group were excluded from analysis. Both groups achieved their feed targets. Peak plasma insulin concentrations (mean ± SD) were significantly higher in the intermittent group versus continuous (295.1 ± 167.8 vs. 128.1 ± 57.2 pmol/L, p < 0.001). Plasma glucose concentrations were not significantly different between groups. There were no between-group differences in other plasma metabolites and there were no adverse events such as hyper-/hypo-glycaemia. There was evidence of increased bowel motility in the intermittent group.

**Conclusion:**

Intermittent diurnal feeding, compared to continuous feeding, preserves the physiological insulin response in critically ill adults. Both regimens were well tolerated, supporting the need for a larger trial to assess other clinically important patient-centred outcomes.

**Trial registration:**

This trial was registered prospectively at clinicaltrials.gov (study ID NCT06115044).

## 1 Introduction

Approximately 200,000 patients are admitted to critical care units in the UK every year [1]. In line with international guidelines, approximately 50 % of these patients are supported with early enteral nutrition via a nasogastric tube, as they will be unable to feed themselves for a prolonged period [2]. Despite known benefits of enteral nutrition in the prevention of catabolism and improved clinical outcomes, the optimal feeding pattern remains uncertain, and nutritional targets are often missed [3,4]. Historically, most critical care units deliver enteral nutrition as a continuous infusion, although this is unphysiological, failing to align with circadian/diurnal rhythms in both human behaviours (i. e. typical meal patterns) and metabolic responses [5].

Intermittent feeding, where feed is delivered over periods of 20–60 min with fasting intervals in between, has been proposed as an alternative to continuous feeding [6]. International guidelines in favour of continuous feeding are largely founded upon widely accepted practice, and an absence of conclusive evidence for the benefits of intermittent feeding in the Intensive Care Unit (ICU) plus a potential signal from a few small studies that intermittent feeding may increase gastrointestinal intolerance, aspiration, and dysglycaemia [7,8]. More recent reviews of the evidence have found no significant differences in aspiration; gastric residual volumes and glucose variation, but highlight a trend towards diarrhoea in intermittent feeding and constipation in continuous feeding groups, respectively [9–11]. The studies included in these reviews show considerable heterogeneity in the feeding regimens, such as the inclusion of nighttime feeding in intermittent arms, which may negate any metabolic benefits of a prolonged overnight fast.

There are other, potentially more compelling, benefits to intermittent feeding. Aligning feeding with wake cycles and fasting with sleep (diurnal feeding), may offer metabolic advantages. Timing of feeds can influence circadian regulation, as synchronisation of nutritional intake with central genetic clocks may alter metabolism of nutrients by peripheral tissues [12,13]. Indeed there is evidence that the timing of feeds can act as a zeitgeber in its own right – so aligning the circadian and metabolic rhythms [14]. In the longer term, misalignment of internal clocks increases the risk of immune dysfunction, insulin resistance and cardiovascular disease [15–18]. In addition, intermittent feeding may better support muscle protein synthesis, enhance gut motility, and better maintain glycaemic control and/or insulin sensitivity [19–22]. Importantly, intake of normal meals in healthy subjects promotes pulsatile release of insulin, a process critical for net anabolism; regulation of glucose, lipid and protein metabolism; and skeletal muscle autophagy and glycaemic control: we don’t know if this is also true in critical illness [23,24]. Pragmatically, intermittent feeding may improve mobility by reducing the need to be connected to a feeding line, and reduce the impact of feed interruptions to help achieve nutritional targets more effectively than continuous feeding [25]. In combination, this could have important impacts in improving metabolic, hormonal, circadian and long-term functional outcomes.

The aim of the “Does Intermittent Nutrition Enterally Normalise hormonal and metabolic responses to feeding in critically ill adults?” (**DINE-Normal)** study was to evaluate both the feasibility and the metabolic and hormonal effects of diurnal intermittent versus continuous feeding in critically ill adult patients. The primary outcome was peak plasma insulin within 3 h of an intermittent feed compared to continuous feeding.

## 2 Methods

### 2.1 Ethics and Trial Registration

This trial was registered prospectively with a Clinical Trials Registry (clinicaltrials.gov - NCT06115044). We obtained ethical approval from the Wales Research Ethics Committee 3 prior to data collection (reference 23/WA/0297). A research-without-prior-consent model was used. This approach was deemed appropriate in patient and public consultation and approved by the Research Ethics Committee. Informed consent was sought once patients regained capacity. Further details are in the published trial protocol [26].

### 2.2 Study design

This study was a prospective, parallel group, randomised, open-label trial. This was a single-centre study, conducted in a 48-bed mixed ICU, set within a 996-bed teaching hospital and major trauma centre.

### 2.3 Participants

Adult patients anticipated to require gastric enteral nutrition for >48 h were eligible to participate. Participants were excluded if any of the following applied: anticipated to be on enteral nutrition for <48 h; requiring parenteral or jejunal nutrition; trophic feed only (e.g., lactate >4); deemed to be at high risk of refeeding syndrome; previous gastrointestinal surgery or pathology; diabetic emergency; pregnancy or prone positioning.

Recruitment took place between December 2023 and April 2024. Participants were screened for eligibility 7 days of the week and could be recruited within 24 h of starting enteral nutrition by an appropriately trained healthcare professional on the study delegation log. Participants were monitored closely for the core study outcomes for 48 h after study entry (trial intervention period), and length-of-stay outcomes were recorded until hospital discharge. The trial ended on completion of the follow-up of the final participant.

### 2.4 Randomisation and blinding

The randomisation sequence was generated using the National Cancer Institute Clinical Trial Randomisation Tool and placed into sealed opaque envelopes by an individual independent to the study prior to enrolment. Patients were randomised in a 1:1 ratio to the intermittent diurnal or continuous feeding trial arms by a member of the study team. Following randomisation, the study was open label, with only the study statistician blinded to group allocation. An open-label trial design was chosen due to both the difficulty of masking the pattern of feed administration, and to allow early identification of possible adverse effects.

### 2.5 Trial intervention

**Fig. 1:**
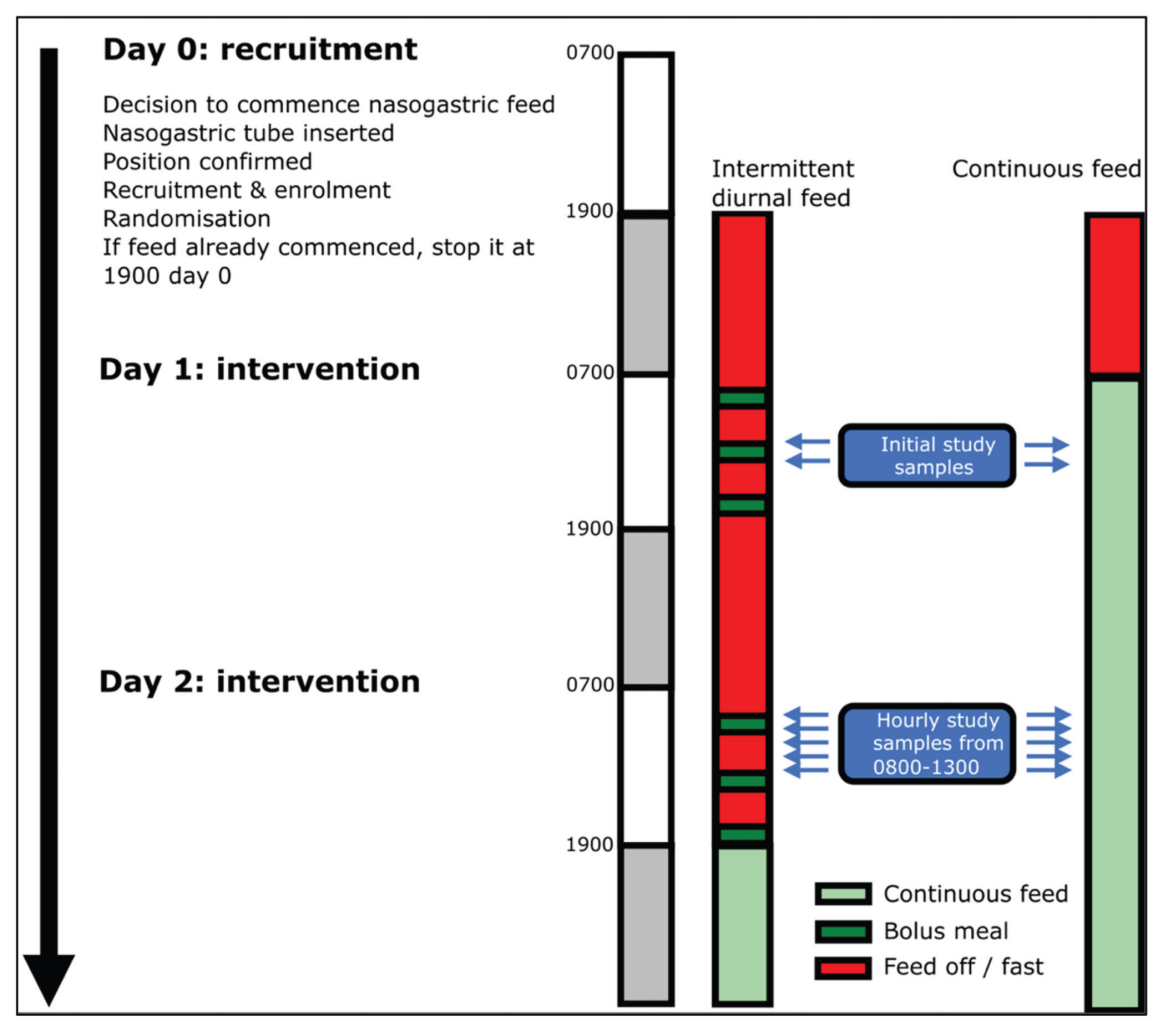
Feeding and sampling timeline.

A detailed description of the trial intervention has been published previously [26]. In brief, the intervention was an adjustment in the delivery of gastric feed, to an intermittent diurnal pattern, for a period of 48 h (Fig. 1).

The intermittent feeds (each one third of the calculated daily feed requirement) were administered at 8:00, 13:00 and 18:00 each day. The comparator group followed a continuous nasogastric feeding regime, as per local nutrition guidelines, to deliver the calculated daily feed requirement (see Supplementary Material). At the end of the 48-h intervention period, patients reverted to the local continuous enteral feeding regimen.

### 2.6 Outcomes

The primary outcome was peak plasma insulin reached within 3 h of a feed compared to the peak over the equivalent time period in the continuous feed group. This was measured for the morning feed on day 2 of the study. Blood samples were taken hourly between 8:00 and 13:00 from an indwelling intra-venous/intra-arterial catheter to allow identification of any peak in plasma insulin levels (Fig. 1).

Secondary outcomes included endocrine and metabolic measures and assessment of feasibility, tolerability and efficacy of the intervention. Planned endocrine and metabolic outcomes assayed from blood plasma included: c-peptide glucose; ketones (beta-hydroxybutyrate); urea; non-esterified fatty acids; triglycerides and glycerol. Feasibility outcomes were: percentage of target nutrition achieved (per 24-h period); absolute calories delivered and protocol compliance. Tolerability outcomes were episodes of vomiting/24-h period; episodes of aspiration of feed; delayed gastric emptying (defined as gastric residual volume >250 ml × 2 in a 24-h period) [27]; ileus; diarrhoea (passage of type 6 or 7 stool according to the Bristol Stool Chart or >3 stool/24 h) and constipation (defined for this study as absence of bowel opening during the 48hr study period) [28]. Indications of efficacy were based on ICU and hospital length of stay; ICU and hospital mortality; Delta-SOFA (Sequential Organ Failure Assessment) score between day 0 and day 2.

### 2.7 Study procedures

Participants were recruited on study day 0, initiating an over-night fast from 19:00 on that day, marking the start of the 48-h intervention period. For the diurnal intermittent group, three feeds were administered each day via a volumetric pump over a period of 30–60 min. On study day 1, each feed consisted of 200 ml (600ml/24 h). On study day 2, each feed provided one third of the individual’s daily caloric requirements, determined using a weight-based equation set by intensive care dieticians in accordance with the local nutrition guideline (see Fig. S1 in Supplementary Material) [8]. Patients in the continuous feeding group had an identical feed amount calculated and delivered as a steady infusion over each 24 h. Patients in the intermittent diurnal feeding group restarted the usual local continuous enteral feeding regimen at 12:00 on day 3 to prevent overfeeding. Patients were monitored within the study for an additional 12 h after the 48-h intervention period to identify any adverse effects potentially attributable to the intervention. The feed type used was Nutrison Protein Plus (Nutricia, UK).

The protocol paper provides further details on: concomitant medication; assessment of compliance; blood sampling and storage; analysis; participant study exit criteria; data collection/management/analysis and monitoring [26].

### 2.8 Biochemical analysis

Plasma insulin was quantified using an enzyme-linked immunosorbent assay (Mercodia, Sweden). Plasma glucose, non-esterified fatty acids (NEFA), glycerol, triglycerides, and urea were determined using commercially available spectrophotometric assays (Randox, UK). The assay for c-peptide failed quality control, therefore no results are available for this analyte.

### 2.9 Statistical analysis

The statistical analysis plan was published a priori in the protocol paper [26]. The desired sample size of 30 participants was based on findings from a study of healthy individuals, where the intermittently fed group had a mean (±SD) peak plasma insulin concentration of 373 ± 204 pmol/L 2 h after feeding, compared to 58 ± 41 pmol/L in the continuously fed group [29]. This standardised effect size of 2.14 indicated that 6 participants per group would provide 90 % power (p = 0.05). Anticipating a smaller effect size in the critically ill population due to reduced feed volumes and impaired physiological capacity to mount a response, the target sample size was adjusted to detect an effect size of 1.26, requiring 11 participants per group for 80 % power. To account for a 25 % dropout rate, the sample size was increased to 30 participants.

Data were analysed by a blinded statistician using an intention-to-treat analysis set. Two-sided statistical tests were used throughout, p < 0.05 taken as statistically significant. The primary outcome was peak plasma insulin within 3 h of the morning intermittent feed on the second study day, compared to continuously fed patients at the equivalent time point. The primary and secondary hormonal and metabolic indicators collected over the study duration were analysed by linear mixed effects modelling including a group by time interaction with post hoc t-testing where appropriate.

Length-of-stay outcomes were compared using Kaplan–Meier analyses and the log-rank test. The percentage achieving target nutrition (per day) was reported with 95 % confidence interval for between-group differences.

## 3 Results

**Fig. 2:**
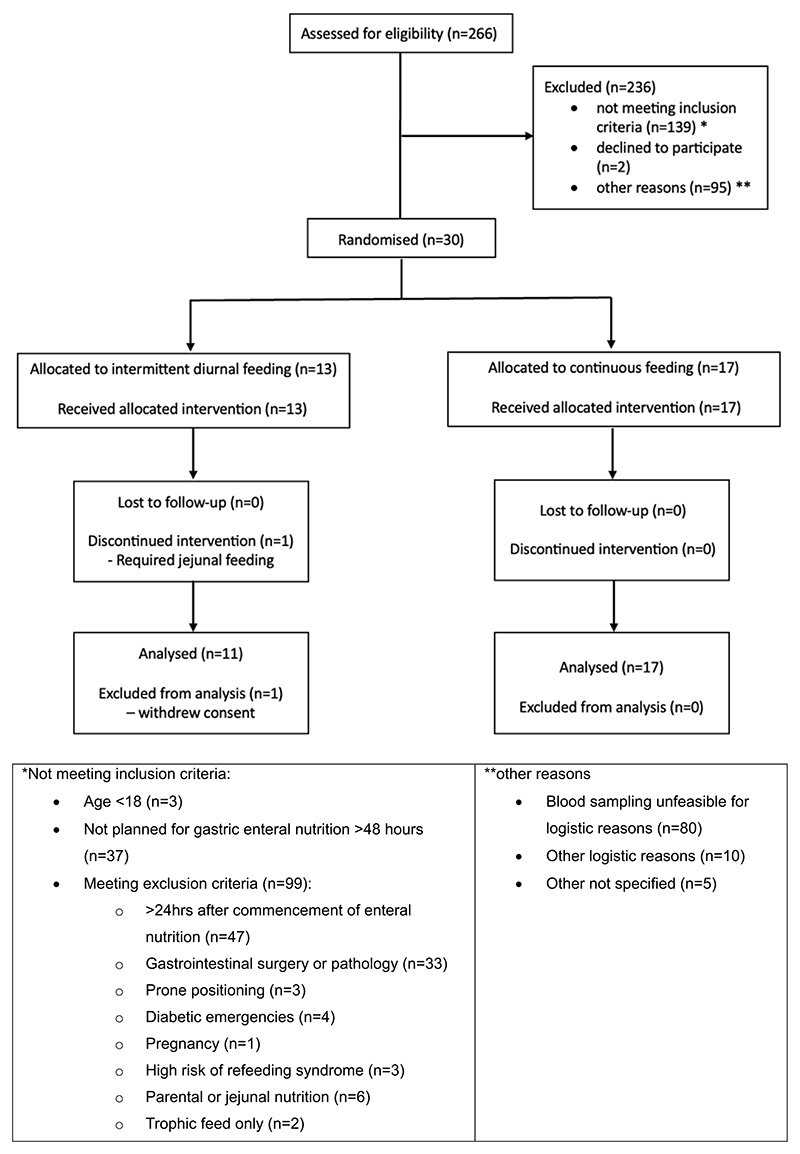
CONSORT diagram showing flow of participants through the trial.

A total of 266 patients were screened between December 2023 and April 2024. Of these, 30 patients were enrolled; 13 patients were randomised to intermittent diurnal feeding and 17 to continuous feeding (Fig. 2). In the intermittent diurnal feeding arm, one patient required jejunal feeding and therefore had to discontinue the study intervention, and one patient withdrew consent. The final analysis included 11 patients in the intermittent diurnal feeding group and 17 patients in the continuous feeding group.

Clinical and demographic characteristics were well matched at baseline (Table 1). There were 2 participants with diabetes in the continuous feeding group; both participants had diet-controlled type 2 diabetes and were not on any oral hypoglycaemic agents or insulin upon enrolment to the study.

**Fig. 3:**
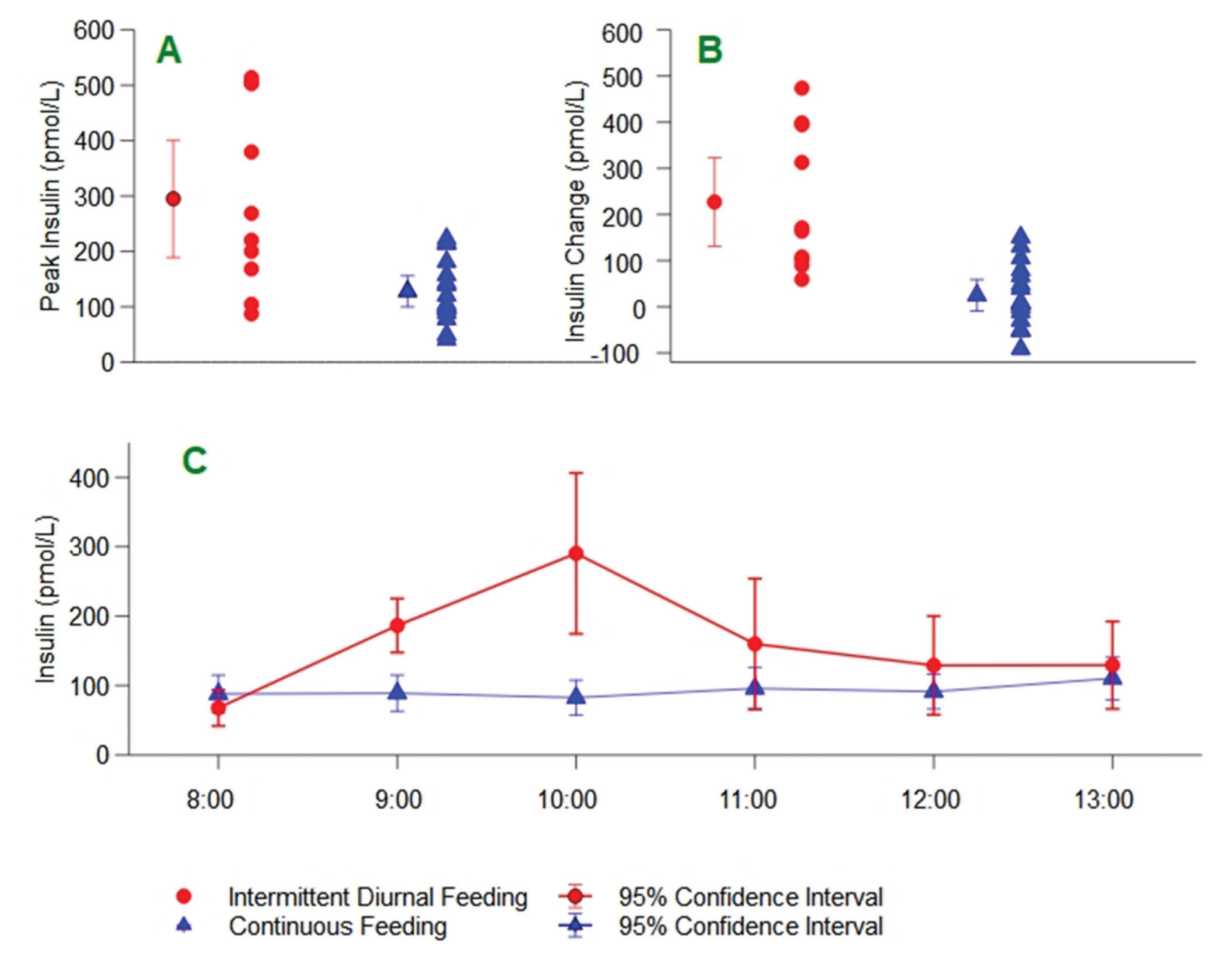
Plasma insulin during the hourly sampling period on study day 2 A: mean peak plasma insulin between 0800 and 1100 study day 2; B: mean increase in plasma insulin from 0800 baseline study day 2; C: Plasma insulin hourly during sampling period.

Peak plasma insulin was significantly higher in the intermittent feeding group versus continuous (295.1 ± 167.8 pmol/L vs. 128.1 ± 57.2 pmol/L, mean ± SD, p < 0.001) (Fig. 3A and Supplementary Table 1), This was also shown by the greater maximum increase from baseline in the intermittent group (Fig. 3B) with a clear peak noted 2 h after the start of the intermittent feed (Fig. 3C).

**Fig. 4:**
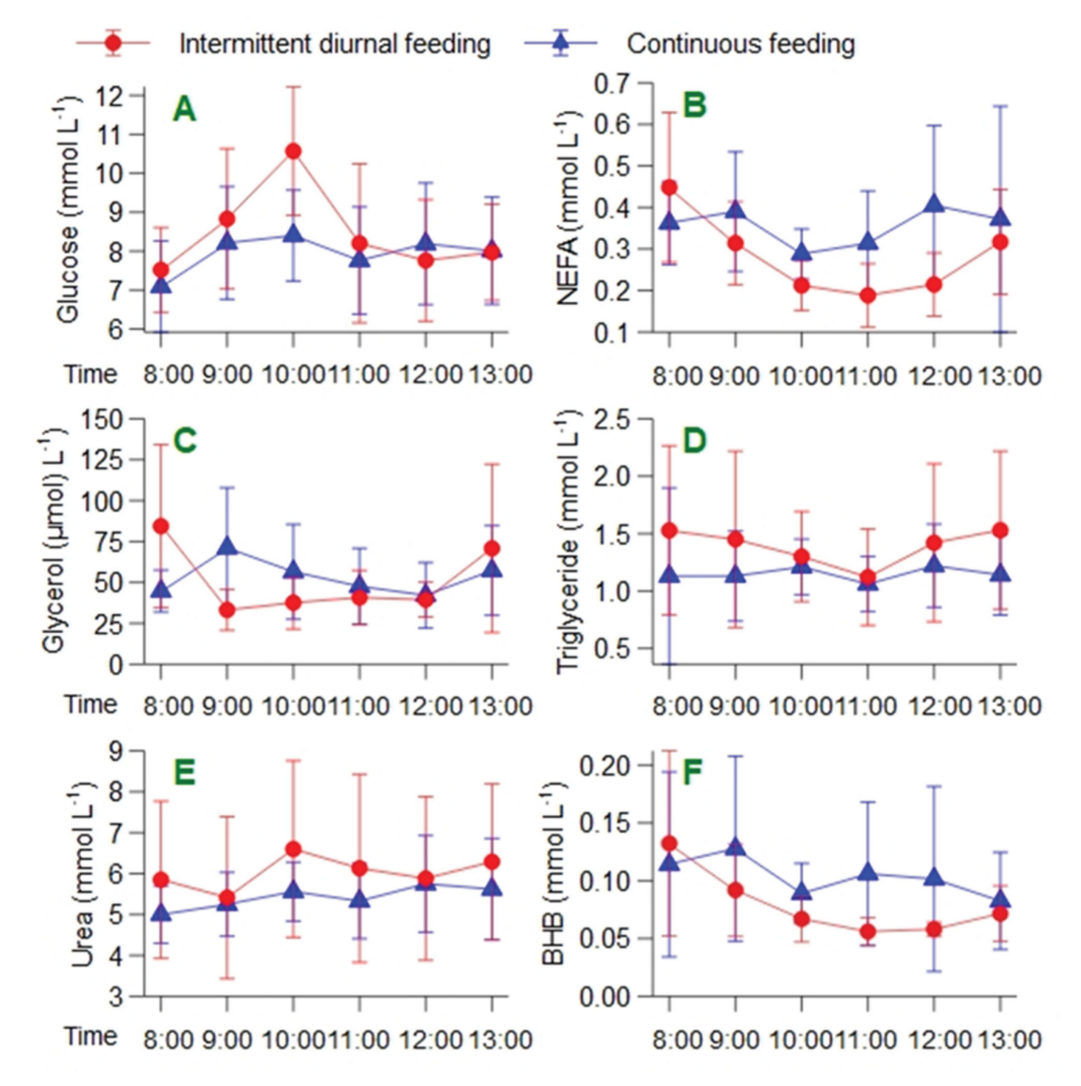
Plasma metabolites during the hourly sampling period on study day 2 A) Glucose concentrations showed a significant rise at 10:00 in the intermittent group paralleling the peak in insulin (p = 0.04). There was a pattern of a reduction in NEFA, glycerol and BHB concentrations across time in the intermittent group (B, C and F, non-significant) without evidence of changes in triglyceride or urea concentrations (D and E). BHB: beta-hydroxybutyrate; NEFA: non-esterified fatty acids.

The difference between groups remained significant in *post hoc* exploratory sensitivity analysis which excluded a single patient who was commenced on an exogenous insulin infusion (in intermittent diurnal feeding group, see Fig. S2 in Supplementary Material) and also when both patients with pre-existing diabetes were excluded. Plasma glucose concentrations during the sampling period varied significantly with time, but this was not dependent on group (Supplementary Table 1). The elevation in the intermittent group at 10:00 (mean ± SD 10.6 ± 2.5 mmol/L vs. 8.4 ± 2.4 mmol/L, p = 0.04, Fig. 4A) was non-significant after correction for multiple comparisons. There were no episodes of hyper- or hypoglycaemia in either group and glucose variability (defined as the difference between the highest and lowest glucose recordings) was higher in the intermittent group on day one but was comparable on day two (Table 2).

Both groups met caloric targets during the study period and no adverse events were recorded. There was no increase in gastric stasis, vomiting, aspiration or ileus in the intermittent group (Table 2). A higher percentage of patients in the intermittent group opened their bowels compared to the continuous group (73 % vs. 47 %) during the 48-h study period (Table 2). Patients in the intermittent feeding group had more frequent bowel movements over the 48-h compared to the continuous feeding group (median (IQR) 2 (0.5–2.5) vs. 0 (0–2)). Diarrhoea (loose or liquid stool) was more frequent in the intermittent feeding arm compared to continuous (5/11 vs. 0/17 patients, p = 0.005). There were no statistically significant between-group differences in the analysis of the metabolites (after correction for multiple comparisons) except for a time-dependent difference in glycerol (Fig. 4 and SupplementaryTable 1). There was no significant difference between the groups in ICU or hospital length of stay (Table 2 and Supplementary Fig. 3).

## 4 Discussion

This study examined the feasibility and effects of a novel regimen of diurnal intermittent enteral feeding compared to continuous enteral feeding on hormonal and metabolic markers in a critically ill population. Our main finding is that intermittent diurnal nutrition produces a postprandial insulin response in critically ill adults similar in timing and magnitude to that seen in healthy adult volunteers [29]. Glucose concentration does not differ between groups or in the incidence of hyper- or hypoglycaemia. Other recent metabolic studies of intermittent feeding in critically ill adults did not report on the endogenous insulin response [30,31].

The intermittent feeding regimen was shown to be feasible and provided comparable calorie delivery to standard continuous feeding without any increase in the incidence of delayed gastric emptying, vomiting, aspiration and with an indication that it may improve bowel motility. From an economic perspective there would be few costs associated with implementation, given the intervention only adjusts the timing of feed delivery. In this our findings are in line with those of other investigators, who also conclude that intermittent feeding is feasible and safe [32].

### 4.1 Insulin

Insulin is a key regulator of metabolism. An increase in insulin in response to feeding will promote cellular glucose uptake and use, along with skeletal muscle amino acid uptake and protein synthesis, with a net anabolic effect. There may also be other direct effects on vital organs such as brain, kidney and endothelium that are all important in critical illness [33].

Additionally, the phasic nature of the insulin peaks and troughs are thought to be an intrinsic component of cell signalling that is crucial for usual homeostatic effects of insulin and that cannot be replicated with continuous feeding [34]. The insulin response to glucose is graded, with β-cells exhibiting a threshold-dependent secretion pattern [35,36]. Acute increases in glucose levels, such as those seen with intermittent feeding, are more likely to trigger significant insulin release compared to the gradual nutrient delivery associated with continuous feeding, which may not reach the necessary threshold for substantial insulin secretion.

Following an overnight fast, insulin concentrations are low which permits increased rates of lipolysis in adipose tissue to provide circulating fatty acids as fuel [37]. This is typically reflected by high circulating concentrations of glycerol and non-esterified fatty acids. In the postprandial state, increased insulin concentrations contribute to controlling glucose concentrations both directly (via suppression of endogenous glucose production and stimulation of peripheral glucose uptake) and indirectly, via suppression adipose tissue lipolysis and thereby reduced competition for mitochondrial substrate oxidation between glucose and fatty acids [38]. The insulin induced suppression of lipolysis also reduces substrate availability for ketogenesis [39]. The overall picture of the hormonal and metabolic patterns observed in the intermittent group in this study (low insulin, with high glycerol and fatty acid concentrations in the fasted state, and suppressed glycerol, non-esterified fatty acid and beta-hydroxybutyrate concentrations in the fed state, without evidence of increases in triglycerides or urea concentrations) appear consistent with this, and may lend support to the argument that this intermittent feeding regimen preserves a more “normal” metabolic and hormonal pattern than does continuous feeding in critically ill patients. Whether this holds for longer periods of intermittent feeding and in larger groups of patients, and whether any difference translates into clinical benefit, are questions that remain to be answered.

### 4.2 Tolerability

This intermittent diurnal feeding intervention was well tolerated. Although we noted a peak in plasma glucose after an intermittent feed, there was no additional risk of hyper- or hypoglycaemia and no increase in the glucose variability, likely reflecting the increased secretion of insulin and other gut hormones promoting glucose uptake and utilisation. One previous meta-analysis highlighted an increased risk of gastrointestinal intolerance and aspiration compared to continuous feeding [40], but this was not observed in our study where there was no increase in gastric stasis, vomiting, aspiration or ileus. We observed an increased incidence of diarrhoea in the intermittent diurnal feeding arm. We defined diarrhoea as passage of type 6 or 7 stool according to the Bristol Stool Chart, or >3 stool/24 h – all of our patient’s diarrhoea diagnoses were on the basis of the presence of loose stool rather than frequency. Indeed, some studies define diarrhoea simply as the passage of >5 stools per day [41], so by this criterion our result represents an overestimate of the incidence of diarrhoea. Given that constipation is a problem for over half of critically ill patients in the first week of ICU admission, intermittent feeding may actually represent maintenance or restoration of normal gut motility, given the higher rates of bowels opening in the intermittent feeding group [42].

### 4.3 Generalisability and sources of bias

Our study has several limitations. Inherent to the design is a lack of blinding that may introduce bias, although this is mitigated somewhat by the objective nature of the primary and secondary outcomes. Blinding was maintained for both the plasma metabolite/hormonal assays and the statistical analysis of all measures. In a definitive study it may be possible to maintain blinding of the study teams by having pre-programmed pump settings and concealment of the displayed rates, although that would compromise patient mobility benefits. In addition, this was a single centre study. Feeding guidelines and practices may vary between Intensive Care Units, as may case mix. However, given the primary outcome is measuring a fundamental physiological response we suggest that the findings are likely to be generalisable and justify further investigation in multiple centres. The sample size is small and the period of intervention short reflecting the early-phase nature of the study but was calculated prospectively against a key hormonal outcome (pulsatile insulin release) with allowance for both a smaller effect size and for drop out.

### 4.4 Specific trial limitations

We were unable to account for non-nutritional calories, including the contribution of lipid-soluble medications or intravenous dextrose. Although theoretically balanced between groups due to randomisation, this may be worth measuring as a potential confounding variable in future large-scale studies. To account for the loss of c-peptide, we ran a post-hoc sensitivity analysis excluding the only patient on exogenous insulin (in the intermittent diurnal group), which made no difference to the primary outcome.

By chance the study included two patients with diabetes, who were managed with diet alone. Sensitivity analysis excluding these two patients did not change the findings of the study. Whether the insulin response to intermittent feeding would be similar in critically ill diabetic patients, and whether there would be any difference between type 1 and type 2 diabetes, will require further investigation.

To avoid harm to the participants from unnecessary oversampling, and due to the resource-intensive nature of blood sampling; we chose to focus intensive blood sampling around the morning feed on study day 2. As a result, there is a chance that we have failed to detect other metabolic phenomena that may have occurred during the study period. We also are unable to comment on any metabolic changes that occur after the study period. Nevertheless, demonstrating an insulin response to intermittent feeding that is both different to that seen with continuous feeding and similar to that in health is consistent with a genuine metabolic phenomenon that has the potential to influence the course of critical illness.

## 5 Conclusion

Diurnal intermittent feeding, compared to continuous feeding, produces a post-prandial insulin response and is consistent with a more physiological metabolic milieu in critically ill adults, without additional risk of dysglycaemia. This was an efficacious and well-tolerated intervention in the ICU which offers other pragmatic benefits, such as improved bowel motility and fewer restrictions on patient mobility. Further research is needed to assess other clinically important outcomes including on long-term survival and function. Future work should also address acceptability of intermittent feeding to patients and professionals and consideration of the cost-effectiveness of the intervention.

## Supplementary Material

Appendix A. Supplementary data

Supplementary data to this article can be found online at https://doi.org/10.1016/j.clnu.2025.10.003.

Supplementary Material

## Figures and Tables

**Table 1:** Baseline characteristics.

	Intermittent diurnal feeding (n = 11)	Continuous feeding (n = 17)
Sex (male)	6/11 (54.5 %)	10/17 (58.8 %)
Age (yrs)	60 (54–70)	60 (54–72)
Mass (kg)	77 (65–83)	75 (67–83)
Height (cm)	174 (165–181.5)	175 (165–182)
Ethnicity	3/11 (27 %) - English, Welsh, Scottish, Northern Irish or British	7/17 (41 %) - English, Welsh, Scottish, Northern Irish or British
	1/11 (9 %) Black, Black British, or Caribbean background	1/17 (6 %) - Black, Black British, or Caribbean background
	7/11 (63 %) – any other ethnic group	9/17 (53 %) – any other ethnic group
SOFA score	7 (5.25–9)	7 (6–9)
ICNARC score	17.5 (14.25–21)	17 (14.75–21.25)
APACHE II score	13 (10.25–21.25)	15 (10.5–22)
Presence/absence of diabetes	0/11	2/17 (both type 2) - 12 %
Medication (insulin, oral hypoglycaemic agents)	No insulin/hypoglycaemic agents	No insulin/hypoglycaemic agents
Time from admission to ICU to start of enteral feeding (hours	11.5 (9.8–31.4)	15.3 (7.6–17.9)
Time from start of enteral feeding to enrolment into study (hours)	23.3 (18.6–33.5)	31.4 (22.9–35.3)

Data are shown as median (IQR) or frequencies (n, (%)). There were no statistically significant differences between the groups. APACHE II: Acute Physiologic Assessment and Chronic Health Evaluation; ICNARC: Intensive Care National Audit and Research Centre; ICU: intensive care unit; IQR: interquartile range; SOFA, Sequential Organ Failure Assessment.

**Table 2:** Secondary Outcomes relating to feasibility and efficacy.

	Intermittent diurnal feeding (n = 11)	Continuous feeding (n = 17)	p-value
%target nutrition day 1	100 (100, 100)	100 (97, 100)	0.890
%target nutrition day 2	100 (100, 100)	105 (92, 110)	0.430
Absolute calories day 1	750 (750, 750)	750 (729, 750)	0.890
Absolute calories day 2	1376 (1249, 1376)	1446 (1266, 1452)	0.285
Bowels not open	3/11 (27)	9/17 (53)	0.253
Diarrhoea	5/11 (45.5)	0/17 (0)	0.005
No. of episodes bowel opening over study period	2 (0.5, 2.5)	0 (0, 2)	
Aspiration	0/11 (0)	0/17 (0)	1.000
Ileus	0/11 (0)	0/17 (0)	1.000
Vomiting	0/11 (0)	2/17 (11.8)	0.505
Delayed gastric emptying	1/11 (9.1)	3/17 (17.6)	0.635
Episodes of hyper or hypoglycaemia	0/11 (0)	0/17 (0)	1.00
Highest glucose day 1	9.7 (8.6, 10.5)	8.4 (7.5, 9.0)	0.053
Highest glucose day 2	9.4 (8.0, 11.3)	9.1 (8.2, 10.2) Not recorded (n = 1)	0.980
Glucose variability (highest-lowest glucose day 1)	2.7 (2.3, 4.7)	1.5 (1.0, 2.7) Not recorded (n = 1)	0.017
Glucose variability (highest-lowest glucose day 2)	2.2 (1.4, 3.8)	2.2 (1.8, 3.3) Not recorded (n = 1)	0.767
No. of patients on exogeneous insulin infusion during study period	1/11	0/17	0.393
ICU LOS, days	8 (10, 21)	13 (10, 22)	0.445
Hospital LOS, days	27 (15, 41)	29 (12, 38)	0.683
ICU mortality	1/11 (9.1)	2/17 (11.8)	0.445
Hospital mortality	3/11 (27)	4/17 (29)	0.683
Delta-SOFA Day 0–2	0 (–2, 3)	0 (–1, +1)	0.781

Data are shown as median (Lower Quartile, Upper Quartile) or frequencies (n, (%)). Day 1 and 2 feeding periods start at 07:00 and run for 24 h (as in Fig. 1). Diarrhoea was defined as passage of type 6 or 7 stool according to the Bristol Stool Chart or >3 stool/24 h. Highest glucose readings on day 2 exclude the period of intensive sampling (reported separately).

ICU: Intensive Care Unit; IQR: interquartile range; LOS, length of stay; SD: standard deviation; SOFA, Sequential Organ Failure Assessment.

## Abbreviations

ICU: Intensive Care Unit
LOS: length of stay
NEFA: non-esterified fatty acids
NG: nasogastric
PPI: Public and Patient Involvement
SOFA: Sequential Organ Failure Assessment
SD: Standard Deviation

## References

[1] Intensive care National Audit & Research Centre (2024). http://icnarc.org.

[2] Binnekade JM, Tepaske R, Bruynzeel P, Mathus-Vliegen EMH, de Hann RJ (2005). Daily enteral feeding practice on the ICU: attainment of goals and interfering factors. Crit Care Lond Engl.

[3] Haines KL, Ohnuma T, Grisel B, Krishnamoorthy V, Raghunathan K, Sulo S (2023). Early enteral nutrition is associated with improved outcomes in critically ill mechanically ventilated medical and surgical patients. Clin Nutr ESPEN.

[4] Bendavid I, Singer P, Theilla M, Themessl-Huber M, Sulz I, Mouhieddine M (2017). NutritionDay ICU: a 7 year worldwide prevalence study of nutrition practice in intensive care. Clin Nutr Edinb Scotl.

[5] McClave SA, Taylor BE, Martindale RG, Warren MM, Johnson DR, Braunschweig C (2016). Guidelines for the provision and assessment of nutrition support therapy in the adult critically ill patient: society of critical care medicine (SCCM) and American Society for parenteral and enteral nutrition (A.S.P.E.N). J Parenter Enteral Nutr.

[6] Ichimaru S (2018). Methods of enteral nutrition administration in critically ill patients: Continuous, cyclic, intermittent, and bolus feeding. Nutr Clin Pract Off Publ Am Soc Parenter Enter Nutr.

[7] Boullata JI, Carrera AL, Harvey L, Escuro AA, Hudson L, Mays A (2017). ASPEN safe practices for enteral nutrition therapy. J Parenter Enteral Nutr.

[8] Singer P, Blaser AR, Berger MM, Alhazzani W, Calder PC, Casaer MP (2019). ESPEN guideline on clinical nutrition in the intensive care unit. Clin Nutr Edinb Scotl.

[9] Mousazadeh N, Hakimi H, Sharif-Nia H, Dorri S (2025). Disadvantages of various methods of gastrointestinal feeding in patients admitted to the intensive care unit: a systematic review. Casp J Intern Med.

[10] Heffernan AJ, Talekar C, Henain M, Purcell L, Palmer M, White H (2022). Comparison of continuous versus intermittent enteral feeding in critically ill patients: a systematic review and meta-analysis. Crit Care Lond Engl.

[11] Qu J, Xu X, Xu C, Ding X, Zhang K, Hu L (2023). The effect of intermittent versus continuous enteral feeding for critically ill patients: a meta-analysis of randomized controlled trials. Front Nutr.

[12] BaHammam AS, Pirzada A (2023). Timing matters: the interplay between early mealtime, circadian rhythms, gene expression, circadian hormones, and Metabolism-A narrative review. Clocks Sleep.

[13] Sagun E, Akyol A, Kaymak C (2024). Chrononutrition in critical illness. Nutr Rev.

[14] Lewis P, Oster H, Korf HW, Foster RG, Erren TC (2020). Food as a circadian time cue - evidence from human studies. Nat Rev Endocrinol.

[15] Boivin DB, Boudreau P, Kosmadopoulos A (2022). Disturbance of the circadian System in shift work and its health impact. J Biol Rhythm.

[16] Stenvers DJ, Scheer FAJL, Schrauwen P, la Fleur SE, Kalsbeek A (2019). Circadian clocks and insulin resistance. Nat Rev Endocrinol.

[17] Alibhai FJ, Tsimakouridze EV, Reitz CJ, Pyle WG, Martino TA (2015). Consequences of circadian and sleep disturbances for the cardiovascular System. Can J Cardiol.

[18] Cuesta M, Boudreau P, Dubeau-Laramée G, Cermakian N, Boivin DB (2016). Simulated night shift disrupts circadian rhythms of immune functions in humans. J Immunol Baltim Md 1950.

[19] Chowdhury AH, Murray K, Hoad CL, Costigan C, Marciani L, Macdonald IA (2016). Effects of bolus and continuous nasogastric feeding on gastric emptying, small bowel water content, superior mesenteric artery blood flow, and plasma hormone concentrations in healthy adults: a randomized crossover study. Ann Surg.

[20] Sjulin TJ, Strilka RJ, Huprikar NA, Cameron LA, Woody PW, Armen SB (2020). Intermittent gastric feeds lower insulin requirements without worsening dysglycemia: a pilot randomized crossover trial. Int J Crit Illn Inj Sci.

[21] Davis TA, Fiorotto ML, Suryawan A (2015). Bolus vs. continuous feeding to optimize anabolism in neonates. Curr Opin Clin Nutr Metab Care.

[22] Gonzalez JT, Dirks ML, Holwerda AM, Kouw IWK, van Loon LJC (2020). Intermittent versus continuous enteral nutrition attenuates increases in insulin and leptin during short-term bed rest. Eur J Appl Physiol.

[23] Cummings DE, Purnell JQ, Frayo RS, Schmidova K, Wisse BE, Weigle DS (2001). A preprandial rise in plasma ghrelin levels suggests a role in meal initiation in humans. Diabetes.

[24] Wilcox G (2005). Insulin and insulin resistance. Clin Biochem Rev.

[25] Hrdy O, Vrbica K, Duba J, Slezak M, Strazevska E, Agalarev V (2025). Intermittent enteral nutrition shortens the time to achieve nutritional goals in critically ill patients. Sci Rep.

[26] Beattie CE, Thomas M, Borislavova B, Smith HA, Ambler M, White P (2024). Does intermittent nutrition enterally normalise hormonal and metabolic responses to feeding in critically ill adults? A protocol for the DINE-Normal proof-of-concept randomised parallel-group study. BMJ Open.

[27] Lew CCH, Lee ZY, Day AG, Heyland DK (2022). Correlation between gastric residual volumes and markers of gastric emptying: a post hoc analysis of a randomized clinical trial. J Parenter Enter Nutr.

[28] Nassar AP, da Silva FMQ, de Cleva R (2009). Constipation in intensive care unit: incidence and risk factors. J Crit Care.

[29] Smith HA, Davis M, Watkins JD, Merrell L, Afman G, Slater T (2023). Effect of nutrient delivery pattern on 24-h rhythms in blood biochemistry, neutrophilic autophagy and the skeletal muscle transcriptome.: 2349. Med Sci Sports Exerc.

[30] Wilkinson D, Gallagher IJ, McNelly A, Bear DE, Hart N, Montgomery HE (2023). The metabolic effects of intermittent versus continuous feeding in critically ill patients. Sci Rep.

[31] Van Dyck L, Vanhorebeek I, Wilmer A, Schrijvers A, Derese I, Mebis L (2020). Towards a fasting-mimicking diet for critically ill patients: the pilot randomized crossover ICU-FM-1 study. Crit Care.

[32] McNelly AS, Bear DE, Connolly BA, Arbane G, Allum L, Tarbhai A (2020). Effect of intermittent or continuous feed on muscle wasting in critical illness. Chest.

[33] Rahman MS, Hossain KS, Das S, Kundu S, Adegoke EO, Rahman MdA (2021). Role of insulin in health and disease: an update. Int J Mol Sci.

[34] Kubota H, Uda S, Matsuzaki F, Yamauchi Y, Kuroda S (2018). In vivo decoding mechanisms of the temporal patterns of blood insulin by the Insulin-AKT pathway in the liver. Cell Syst.

[35] Shankar SS, Shankar RR, Mixson LA, Miller DL, Chung C, Cilissen C (2016). Linearity of β-cell response across the metabolic spectrum and to pharmacology: insights from a graded glucose infusion-based investigation series. Am J Physiol Endocrinol Metab.

[36] Toffolo G, Breda E, Cavaghan MK, Ehrmann DA, Polonsky KS, Cobelli C (2001). Quantitative indexes of beta-cell function during graded up&down glucose infusion from C-peptide minimal models. Am J Physiol Endocrinol Metab.

[37] Ruge T, Hodson L, Cheeseman J, Dennis AL, Fielding BA, Humphreys SM (2009). Fasted to fed trafficking of fatty acids in human adipose tissue reveals a novel regulatory step for enhanced fat storage. J Clin Endocrinol Metab.

[38] Groop LC, Bonadonna RC, DelPrato S, Ratheiser K, Zyck K, Ferrannini E (1989). Glucose and free fatty acid metabolism in non-insulin-dependent diabetes mellitus. Evidence for multiple sites of insulin resistance. J Clin Investig.

[39] Beylot M (1996). Regulation of in vivo ketogenesis: role of free fatty acids and control by epinephrine, thyroid hormones, insulin and glucagon. Diabetes Metab.

[40] Ma Y, Cheng J, Liu L, Chen K, Fang Y, Wang G (2021). Intermittent versus continuous enteral nutrition on feeding intolerance in critically ill adults: a meta-analysis of randomized controlled trials. Int J Nurs Stud.

[41] López-Herce J (2009). Gastrointestinal complications in critically ill patients: what differs between adults and children?. Curr Opin Clin Nutr Metab Care.

[42] Yoshida T, Uchino S, Sasabuchi Y (2022). Epidemiology of constipation in critically ill patients and its impact on in-hospital mortality: a retrospective observational study. J Anesth.

