# Supplementary material for: Does Intermittent Nutrition Enterally Normalise hormonal and metabolic responses to feeding in critically ill adults? The DINE-normal proof-of-concept study
Source: Clin Nutr. Author manuscript; Available in PMC 2026 Mar 28. (PMC7618939; doi:10.1016/j.clnu.2025.10.003)
Supplement: Supplementary Material [file EMS212862-supplement-Supplementary_Material.docx]

Supplementary Material

**D**oes **I**ntermittent **N**utrition **E**nterally **Normal**ise hormonal and metabolic responses to feeding in critically ill adults? The DINE-Normal proof-of-concept study.

Beattie C E, Borislavova B, Smith H A et al.

Table of Contents

| Supplementary figure 1 - Intensive Care Unit Nutrition Guideline | Page 2 |
| --- | --- |
| Supplementary figure 2 - Sensitivity analysis of plasma insulin | Page 3 |
| Supplementary figure 3 - ICU and hospital length of stay | Page 4 |
| Supplementary table 1 - Linear mixed model analysis | Page 5 |


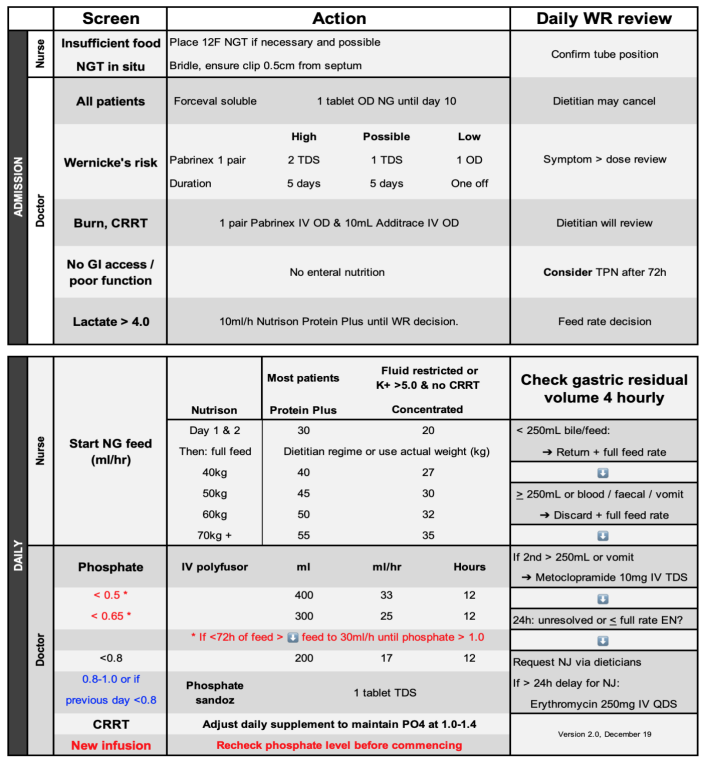


Supplementary Figure 1: summary table of the Intensive Care Unit Feeding Guideline (2022).

CRRT: continuous renal replacement therapy; EN: enteral nutrition; IV: intravenous; K: potassium; NG: nasogastric; NGT: nasogastric tube; NJ: nasojejunal; OD: once daily; PO4: phosphate; QDS: four times daily; TDS: three times daily; TPN: total parenteral nutrition; WR: ward round.


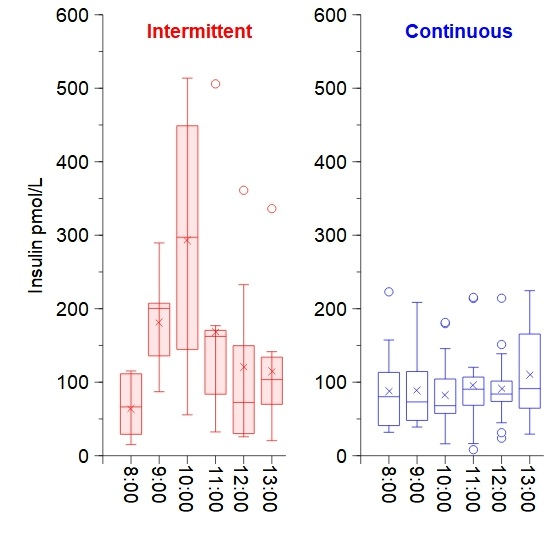


Supplementary Figure 2: Summary distributions of plasma insulin excluding patient on exogenous insulin infusion (n=1). Omission of this patient did not affect the between-group statistical significance at any time point.


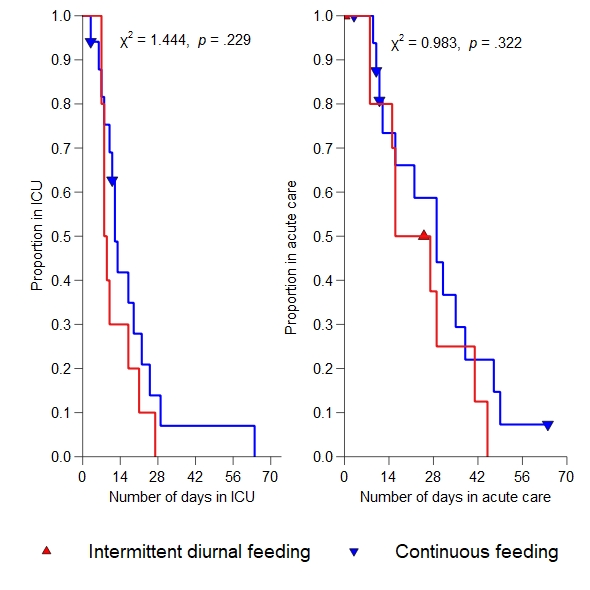


Supplementary Figure 3: Kaplan-Meier survival curve for length of stay in intensive care and acute hospital. A) Total number of days in ICU censored by death are not significantly different (p = .229) B) total number of days in acute care censored by death are not significantly different (p = .322). (log rank Mantel-Cox test, n = 27 - missing data on one; two patients withdrawn).

| Parameter | Main Effect  Randomised Arm | Main Effect  Time | Interaction  Arm by Time |
| --- | --- | --- | --- |
|  |  |  |  |
| Insulin | .004 | < .001 | < .001 |
| Glucose | .491 | <.001 | .095 |
| NEFA | .350 | .154 | .444 |
| Glycerol | .882 | .328 | .035 |
| Triglyceride | .289 | .208 | .641 |
| Urea | .562 | .135 | .480 |
| BHB | .613 | .047 | .419 |
|  |  |  |  |

Supplementary Table 1: Linear mixed model analysis - Randomised arm by time point.
